# Impact of outdoor and nature-based interventions on self-efficacy among adolescents and young adults: a three-level meta-analysis

**DOI:** 10.3389/fpsyt.2026.1856344

**Published:** 2026-07-14

**Authors:** Qiang Zhang, Zhibo Tian, Hongliang Wang, Shiwei Chen, Xue Han

**Affiliations:** 1 College of Physical Education and Health, Guangxi Normal University, Guilin, China; 2 School of Physical Education and Health, Yancheng Normal University, Yancheng, China; 3 School of Sport Science, Beijing Sport University, Beijing, China; 4 Rehabilitation Center, Hebei Institute of Sports Science, Shijiazhuang, China; 5 Key Laboratory of Training Load Diagnosis and Regulation for Elite Athletes, General Administration of Sport of China, Shijiazhuang, China; 6 Hebei Key Laboratory of Digital Physical Fitness Monitoring and Health Promotion, Shijiazhuang, China

**Keywords:** adolescents, outdoor exercise, self-efficacy, three-level meta-analysis, young adults

## Abstract

**Introduction:**

Self-efficacy plays a critical role in motivation, emotional regulation, and adaptive functioning, particularly during adolescence and young adulthood. Outdoor and nature-based interventions have been proposed as a promising approach to enhance self-efficacy. However, prior meta-analyses have been limited by the assumption of independent effect sizes, which may not adequately account for multiple outcomes reported within the same study. This study aimed to systematically evaluate the effects of outdoor and nature-based interventions on self-efficacy among adolescents and young adults using a three-level meta-analytic approach.

**Methods:**

A systematic literature search was conducted in PubMed, Web of Science, Embase, Cochrane Library, SPORTDiscus, and PsycArticles for studies published up to December 8, 2025. Standardized mean differences (Cohen’s d) were calculated using post-intervention means and standard deviations from the intervention and comparison groups. A three-level random-effects meta-analysis was performed to account for statistical dependence among multiple effect sizes. Meta-regression analyses were conducted to examine the potential moderating effects of intervention duration and population type.

**Results:**

Six independent studies comprising 10 effect sizes were included, involving 1,356 participants aged 12–24 years. The three-level meta-analysis demonstrated a significant positive effect of outdoor and nature-based interventions on self-efficacy (d = 0.80, 95% CI 0.31 to 1.30, p = 0.005). Meta-regression analyses indicated no significant moderating effects of intervention duration or population type.

**Conclusion:**

Outdoor and nature-based interventions were associated with improvements in self-efficacy among adolescents and young adults. No significant moderating effects were observed for age group or intervention duration. These findings suggest that outdoor and nature-based interventions may represent a promising approach for supporting self-efficacy during critical developmental periods. However, the findings should be interpreted cautiously given the small number of available studies and indications of potential small-study effects.

**Systematic review registration:**

https://www.crd.york.ac.uk/prospero/, identifier CRD420261293707.

## Introduction

Self-efficacy, a core construct proposed within Social Cognitive Theory, refers to individuals’ beliefs in their capability to organize and execute actions required to manage prospective situations (1). Rather than reflecting actual skills, self-efficacy represents a judgment of what one can do with the skills one possesses (2). Extensive research has demonstrated that self-efficacy plays a critical role in motivation, emotional regulation, behavioral persistence, and performance across a wide range of domains, including education, health behaviors, and psychological well-being (3, 4). Individuals with higher self-efficacy are more likely to set challenging goals, persist in the face of difficulties, and recover more quickly from setbacks, whereas low self-efficacy is associated with avoidance behaviors, reduced motivation, and vulnerability to stress and mental health problems (3, 5).

Adolescence and young adulthood represent critical developmental stages characterized by rapid biological, cognitive, and psychosocial changes, increasing academic and social demands, identity exploration, and heightened exposure to stressors (6, 7). During this period, self-efficacy plays a particularly important role because beliefs about one’s capabilities influence behavioral choices, emotional regulation, health-related decision-making, and long-term developmental trajectories (8). Higher self-efficacy has been associated with better academic achievement, healthier lifestyles, stronger coping abilities, and lower levels of anxiety and depression among adolescents and young adults (9–11). Consequently, identifying effective strategies to enhance self-efficacy during this developmental period is of considerable importance.

Physical activity has long been recognized as an effective means of promoting psychological well-being and strengthening self-efficacy (4). According to Social Cognitive Theory, mastery experiences are the most influential source of self-efficacy beliefs (12), and participation in physical activity provides repeated opportunities for skill development, challenge, goal attainment, and successful performance experiences. Through these experiences, individuals may develop greater confidence in their ability to manage challenges and achieve desired outcomes.

Among physical activity–related approaches, outdoor and nature-based interventions have received growing attention. Previous studies have conceptualized outdoor education, adventure education, wilderness programs, forest schools, and related nature-oriented activities as interventions that intentionally engage participants in structured experiences within outdoor or natural environments to promote learning, health, well-being, and personal development (13–15). Although intervention formats vary, these approaches share a common emphasis on experiential engagement in natural settings. Emerging evidence suggests that nature connectedness, defined as individuals’ subjective sense of connection with the natural environment, may represent an important mechanism through which outdoor and nature-based interventions promote psychological well-being. Greater nature connectedness has been associated with improved mental well-being, lower stress and anxiety, stronger social connectedness, and greater resilience among adolescents and young adults (16–18). Accordingly, strengthening connections with nature may provide an additional pathway through which outdoor and nature-based interventions facilitate positive developmental outcomes, including the enhancement of self-efficacy. Such environments may facilitate self-efficacy development by providing opportunities for mastery experiences, autonomy, problem-solving, social interaction, and exposure to novel challenges (19, 20). Compared with conventional indoor activities, outdoor and natural settings may offer unique experiential contexts that further support perceived competence and personal growth (21, 22). Intervention duration may also influence the effectiveness of outdoor and nature-based interventions, as prolonged participation can provide greater opportunities for experiential learning, skill development, and engagement with natural environments.

Accumulating evidence suggests that participation in outdoor and nature-based interventions is positively associated with self-efficacy among adolescents and young adults (23, 24). However, existing findings remain fragmented and sometimes inconsistent, with variation in intervention characteristics, study designs, participant populations, and outcome measures (25).

In particular, intervention duration has been identified as a potentially important factor influencing the effectiveness of nature-based interventions, although previous findings regarding its relationship with intervention outcomes have been mixed (26, 27). At the same time, Conventional meta-analyses rely on the assumption of independent effect sizes, typically extracting a single effect size from each study (28). However, research on outdoor and nature-based interventions and self-efficacy frequently reports multiple effect sizes derived from the same sample, such as different dimensions of self-efficacy (e.g., general, academic, physical, or task-specific self-efficacy) and multiple assessment time points (e.g., pre- and post-intervention or follow-up measurements) (13, 29). Restricting meta-analyses to a single effect size per study may therefore result in substantial loss of information and limit the ability to fully capture the complexity and variability of the relationship between outdoor and nature-based interventions and self-efficacy.

To address these methodological limitations, the present study employs a three-level meta-analysis approach, which allows for the inclusion of multiple dependent effect sizes while appropriately accounting for their statistical dependence (30). By simultaneously modeling sampling variance, within-study variance, and between-study variance, this approach provides a more precise and comprehensive synthesis of the available evidence (31). Therefore, this study aimed to synthesize the available evidence and examine the overall association between outdoor and nature-based interventions and self-efficacy among adolescents and young adults while appropriately accounting for dependence among multiple effect sizes derived from the same study.

## Methods

The conduct and reporting of this systematic review and meta-analysis were informed by the PRISMA framework (32). The study protocol has been registered with PROSPERO (CRD420261293707).

### Literature search and selection

A systematic literature search was conducted to identify studies examining the effects of outdoor and nature-based interventions on self-efficacy among adolescents and young adults. Searches were performed in the following electronic databases: PubMed, Web of Science, Embase, Cochrane Library, SPORTDiscus, and PsycArticles, covering all records published up to December 8, 2025.

The search strategy was structured in line with a PICO-informed framework, including adolescents and young adults as the population, outdoor and nature-based interventions as the intervention, comparison conditions where applicable, and self-efficacy as the outcome. The search strategy combined three key conceptual components: outdoor or nature-based educational and adventure programs, adolescent and young adult populations, and self-efficacy outcomes. Specifically, the search terms included variations of outdoor education and adventure-based interventions (e.g., outdoor education, adventure education, wilderness programs, outdoor learning, experiential learning, forest schools, Outward Bound, and broader combinations of outdoor or nature settings with educational or programmatic terms), population-related terms (e.g., adolescent, teen, youth, young people, young adult, emerging adult, college student, university student, undergraduate, middle school, high school, and secondary school), and self-efficacy–related terms (e.g., self-efficacy, general self-efficacy, academic self-efficacy). Searches were conducted within titles, abstracts, and keywords.

The three conceptual components were combined using Boolean operators, and only records that simultaneously met all three concept criteria were retrieved. The complete search strategy is provided in **Supplementary File 1**.

### Inclusion and exclusion criteria

Studies were eligible for inclusion if they investigated the effects of outdoor and nature-based interventions on self-efficacy among adolescents or young adults. In this study, adolescents and young adults (AYA) were defined according to the framework proposed by the Lancet Commission on Adolescent Health and Wellbeing, which conceptualizes young people as individuals aged 10–24 years and uses the composite term “adolescents and young adults” to refer to this age range (33). Under this framework, the target population encompasses both adolescence and young adulthood rather than defining the entire age range as young adulthood alone.

In this study, outdoor and nature-based interventions were defined as structured programs delivered in outdoor or natural environments. These interventions may include educational, experiential, adventure-based, therapeutic, or physically engaging components, such as outdoor education, outdoor learning, adventure education, wilderness programs, forest schools, and related organized activities conducted in natural settings.

Eligible studies were required to involve participants classified as adolescents or young adults, implement interventions delivered in outdoor or natural environments consistent with the predefined search strategy (e.g., outdoor education, adventure-based programs, or wilderness-related interventions), and assess self-efficacy as a quantitative outcome using validated measurement instruments. In addition, studies were required to adopt an experimental or quasi-experimental design and report quantitative self-efficacy outcomes following the intervention. To be included in the meta-analysis, sufficient statistical information had to be available to calculate standardized effect sizes, including post-intervention means and standard deviations for both intervention and comparison groups. Control conditions were not restricted to a single format, but studies were required to include a comparison group that did not receive the focal outdoor and nature-based intervention.

Studies were excluded if they did not focus on self-efficacy as an outcome, employed outcome measures that were not comparable across studies, or failed to report sufficient data to permit effect size calculation. Studies using ineligible designs, such as purely observational studies without an intervention, qualitative studies, reviews, or case reports, were also excluded. When multiple reports were based on the same dataset, only the most complete or relevant report was included to avoid duplication.

### Data extraction and coding process

The coding sheet included fields covering study identification, sample characteristics, intervention characteristics, outcome measures, effect size data, and variables relevant to moderator analyses. Data from all eligible studies were extracted using a standardized coding spreadsheet specifically developed by the research team for this meta-analysis. Sample characteristics were extracted, including total sample size, group-specific sample sizes for intervention and control groups, reported age range or mean age, sex distribution (percentage of female participants when available), and age-group classification (<18 years or ≥18 years).

Detailed intervention characteristics were coded for each effect size, including the type of outdoor and nature-based interventions (e.g., wilderness therapy, outdoor education, adventure-based training, hiking, or outdoor adventure programs) and the duration of the intervention. When studies reported multiple post-intervention assessment time points (e.g., immediate post-intervention and follow-up assessments), each time point was treated as a separate effect size and coded accordingly.

Self-efficacy outcomes were extracted based on the specific measurement instruments used in each study. The included instruments comprised the Potency Scale (34), General Self-Efficacy Scale (GSES; 35), New General Self-Efficacy Scale (NGSE; 36), Self-Efficacy Questionnaire (37), and the Review of Personal Effectiveness Scale (ROPE; 38). Although these instruments differed in scope and specificity, they were all designed to assess individuals’ perceived capability, competence, effectiveness, or confidence in managing challenges and achieving desired outcomes. While conceptually distinct, these measures reflect closely related dimensions of self-efficacy and were therefore considered sufficiently comparable for quantitative synthesis. Accordingly, they were synthesized using standardized mean differences. References for all measurement instruments are provided in **Table 1**.

**Table 1:** Characteristics of included studies.

Author	Country	N (total sample)	Measure	Duration (days)	% Female	Age(years)	Intervention
Margalit & Ben-Ari (13)	Israel	50	Potency Scale (34)	4	0	E:15 (0.89) C:15 (0.43)	Wilderness therapy program (backpacking and outdoor challenge activities)
Mutz & Müller (39)	Germany	22	General Self-efficacy Scale (35)	9	41.7	19-24	Outdoor hike program
Deane et al. (14)	New Zealand	899	Self-Efficacy Questionnaire (37)	21	N/A	13–15	Wilderness adventure program (outdoor challenge and personal development activities)
Purdie et al. (40)	Australia	177	Review of Personal Effectiveness scale (38)	6	N/A	12–17	Outdoor education program (adventure-based learning activities)
Hunter et al. (15)	New Zealand	110	General Self-efficacy Scale (35)	10	51.6	16 (0.87)	Outdoor education program (sail-training and leadership development activities)
Tyne et al. (41)	UK	98	New General Self-efficacy Scale (36)	4	48	24 (6.8)	Outdoor adventure program (high ropes and aerial challenge activities)

N, total sample size; E, intervention group; C, comparison group; N/A, not available. Age is reported either as an age range (minimum–maximum years) or as mean (SD), according to the format provided in the original studies. Values presented in parentheses represent standard deviations.

For quantitative synthesis, post-intervention means and standard deviations were extracted for both intervention and comparison groups. Standardized mean differences were calculated based on post-intervention group differences and used as the effect size metric in the meta-analysis. When multiple post-intervention assessment time points were reported, each eligible time point was extracted and treated as a separate effect size.

In addition, variables relevant to moderator analyses were coded *a priori*. Intervention duration was coded as outdoor time. Participant age was coded as a categorical moderator based on mean age. Specifically, studies with a mean participant age below 18 years were assigned to one category, whereas studies with a mean participant age of 18 years or above were assigned to the other category. When age ranges were reported, classification was based on the reported mean age or the predominant age range of participants. These criteria were applied consistently across all included studies. Multiple effect sizes originating from the same study were retained and coded as nested within studies to accommodate the three-level meta-analytic model.

All data extraction and coding procedures were conducted independently by two pairs of reviewers (Q.Z. & Z.T.; W.H. & S.C.). Inter-rater agreement was assessed using percentage agreement across study selection, data extraction, with agreement levels exceeding 90%, indicating a high level of consistency between reviewers. Extracted data were cross-checked for accuracy, and any discrepancies were resolved through discussion and adjudication by the corresponding author (X.H.).

### Risk of bias and publication bias assessment

The methodological quality of the included studies was assessed using design-specific risk-of-bias tools. Randomized controlled trials were evaluated using the Cochrane Risk of Bias 2 (RoB 2) tool, whereas non-randomized intervention studies were assessed using the ROBINS-I framework (42). The assessments considered potential sources of bias related to the randomization process, deviations from intended interventions, missing outcome data, outcome measurement, and selective reporting for randomized studies, as well as confounding, participant selection, intervention classification, deviations from intended interventions, missing data, outcome measurement, and selective reporting for non-randomized studies. Domain-level judgments were made for each study and subsequently synthesized into an overall risk-of-bias classification.

Risk-of-bias assessments were conducted independently by two pairs of reviewers (Q.Z. & Z.T.; W.H. & S.C.). Any disagreements were resolved through discussion, and final decisions were made by the corresponding author (X.H.).

Potential publication bias and small-study effects were examined using funnel plot inspection and a mixed-effects meta-regression approach (43). Funnel plot asymmetry was formally tested by regressing effect sizes on their standard errors within a multilevel modeling framework, thereby accounting for the dependence among effect sizes. To further evaluate the robustness of the pooled effect, the estimated effect size as the standard error approached zero was calculated. This approach provides an adjusted estimate of the intervention effect under the assumption that small-study effects are present.

### Statistical analysis

All statistical analyses were conducted using R (R Foundation for Statistical Computing), primarily with the metafor package (44). Effect sizes were calculated as standardized mean differences (Cohen’s d) based on post-intervention self-efficacy scores, representing the difference between outdoor intervention and comparison groups. Effect sizes were coded such that positive values indicated higher post-intervention self-efficacy in the outdoor exercise group. Sampling variances for each effect size were computed using standard formulas for independent-group standardized mean differences.

### Three-level meta-analysis

Because several primary studies contributed more than one effect size (e.g., multiple outcomes or multiple assessment time points), pooled effects were estimated using a three-level random-effects meta-analytic model to account for the non-independence of effect sizes (28). In this framework, sampling variance was modeled at Level 1, effect sizes were nested within studies at Level 2, and between-study variability was modeled at Level 3. Model estimation was performed using restricted maximum likelihood (REML). Statistical inference was based on t-distributions, which are appropriate when the number of included studies is relatively small (45). Although multivariate meta-analytic estimation routines were used to fit the models, their primary purpose was to accommodate dependent effect sizes rather than to jointly model multiple distinct outcomes.

Overall pooled effect sizes with corresponding 95% confidence intervals were calculated. Statistical heterogeneity was assessed using the Q statistic, and total heterogeneity was further decomposed into within-study and between-study components. The proportion of total variance attributable to each level of heterogeneity was summarized using multilevel I² statistics, alongside the corresponding variance estimates (τ²) (46).

### Moderator analysis

Potential sources of heterogeneity were examined using mixed-effects meta-regression models within the three-level framework (44). Intervention duration was included as a continuous moderator. In addition, categorical moderators were examined, including participant age group based on mean age (<18 years vs. ≥18 years). Moderator effects were evaluated using omnibus Wald-type tests with t-based inference and are reported as F statistics with associated degrees of freedom (47). Regression coefficients and subgroup-specific pooled estimates are presented with 95% confidence intervals. These moderators were selected *a priori* because intervention duration and participant age group were the most consistently reported study-level characteristics and were theoretically relevant to variation in self-efficacy outcomes.

### Sensitivity analysis

To evaluate the robustness of the findings, sensitivity analyses were conducted in two ways. First, leave-one-out analyses were performed by sequentially removing each study and re-estimating the pooled effect size. Second, an additional random-effects meta-analysis including only one effect size per study was conducted to assess whether the overall conclusions were influenced by the inclusion of multiple effect sizes from individual studies.

## Results

### Search results and study characteristics

The literature search included studies available up to December 8, 2025. A total of 1,330 records were identified through electronic databases, including PubMed, Web of Science, Embase, Cochrane Library, SPORTDiscus, and PsycArticles, with an additional seven records identified through other sources, including manual reference screening and website searches, which were conducted to minimize publication bias and ensure comprehensive coverage. After removal of 730 duplicate records, 600 records were screened based on titles and abstracts, of which 560 records were excluded.

**Figure 1:**
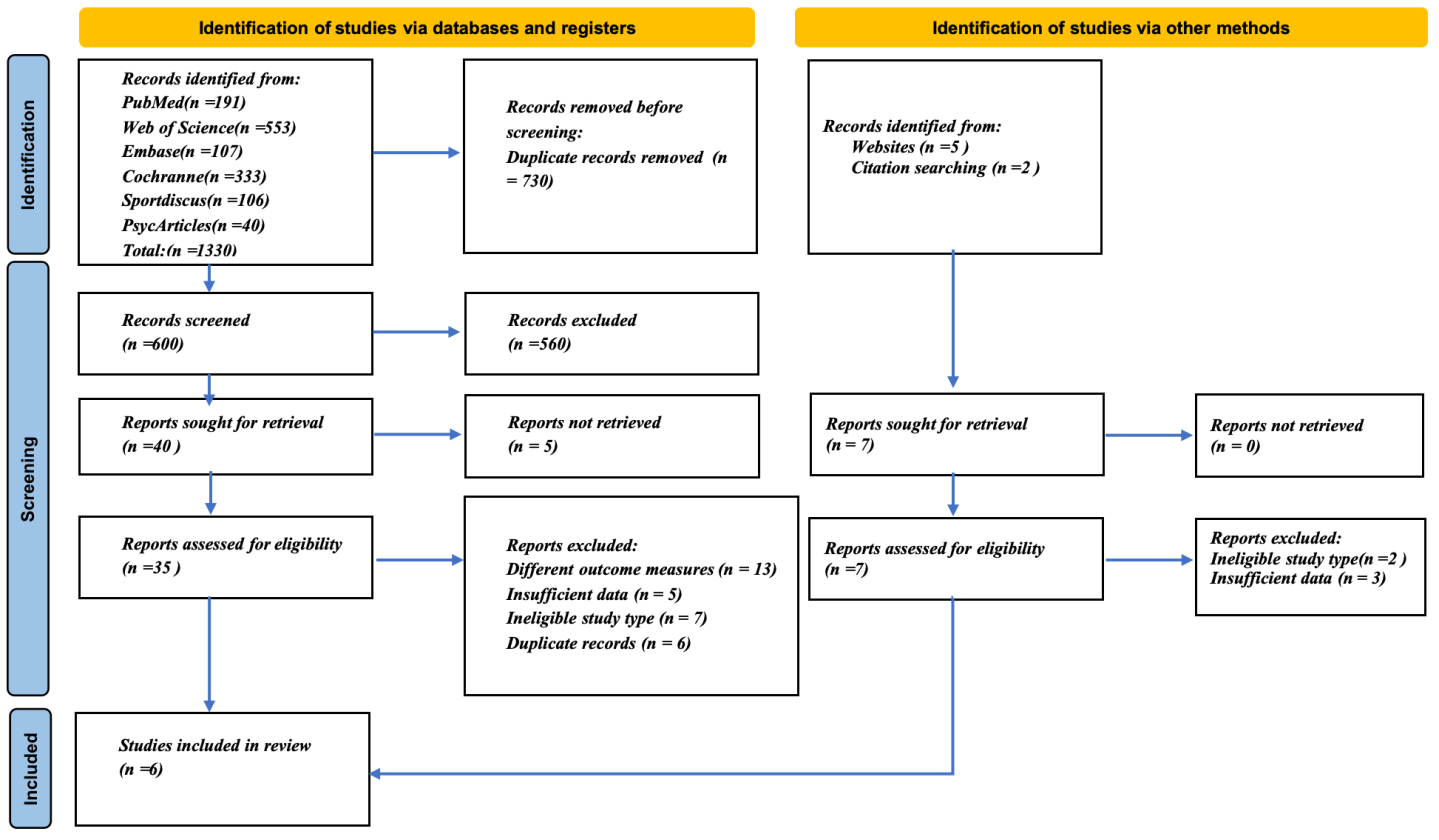
PRISMA 2020 flow diagram illustrating the process of study identification, screening, eligibility assessment, and final inclusion of studies in the meta-analysis.

A total of 47 reports were sought for full-text retrieval. Of these, 5 reports could not be included in the full-text assessment due to unavailability or insufficient information, leaving 42 reports for eligibility assessment. Of these, 34 reports were excluded due to different outcome measures, insufficient data, ineligible study designs, or duplicate records. Ultimately, six studies (13–15, 39–41) met the inclusion criteria and were included in the meta-analysis. The study selection process is illustrated in **Figure 1**.

The six included studies comprised a total sample of 1,356 participants, conducted across five countries, including Israel, Germany, New Zealand, Australia, and the United Kingdom. Sample sizes ranged from 22 to 899 participants. The proportion of female participants ranged from 0% to 51.6%, with several studies not reporting gender distribution.

Participants were adolescents and young adults, with reported age ranges spanning from 12 to 24 years. Intervention durations ranged from 4 to 21 days. A variety of validated self-efficacy measures were used, including the General Self-Efficacy Scale, Potency Scale, and domain-specific self-efficacy questionnaires. Outdoor and Nature-Based Interventions included Wilderness therapy program, Outdoor hike program, Wilderness adventure program, Outdoor education program, Outdoor adventure program. Detailed characteristics of the included studies are presented in **Table 1**.

### Risk of bias and publication bias and sensitivity analysis

**Figure 2:**
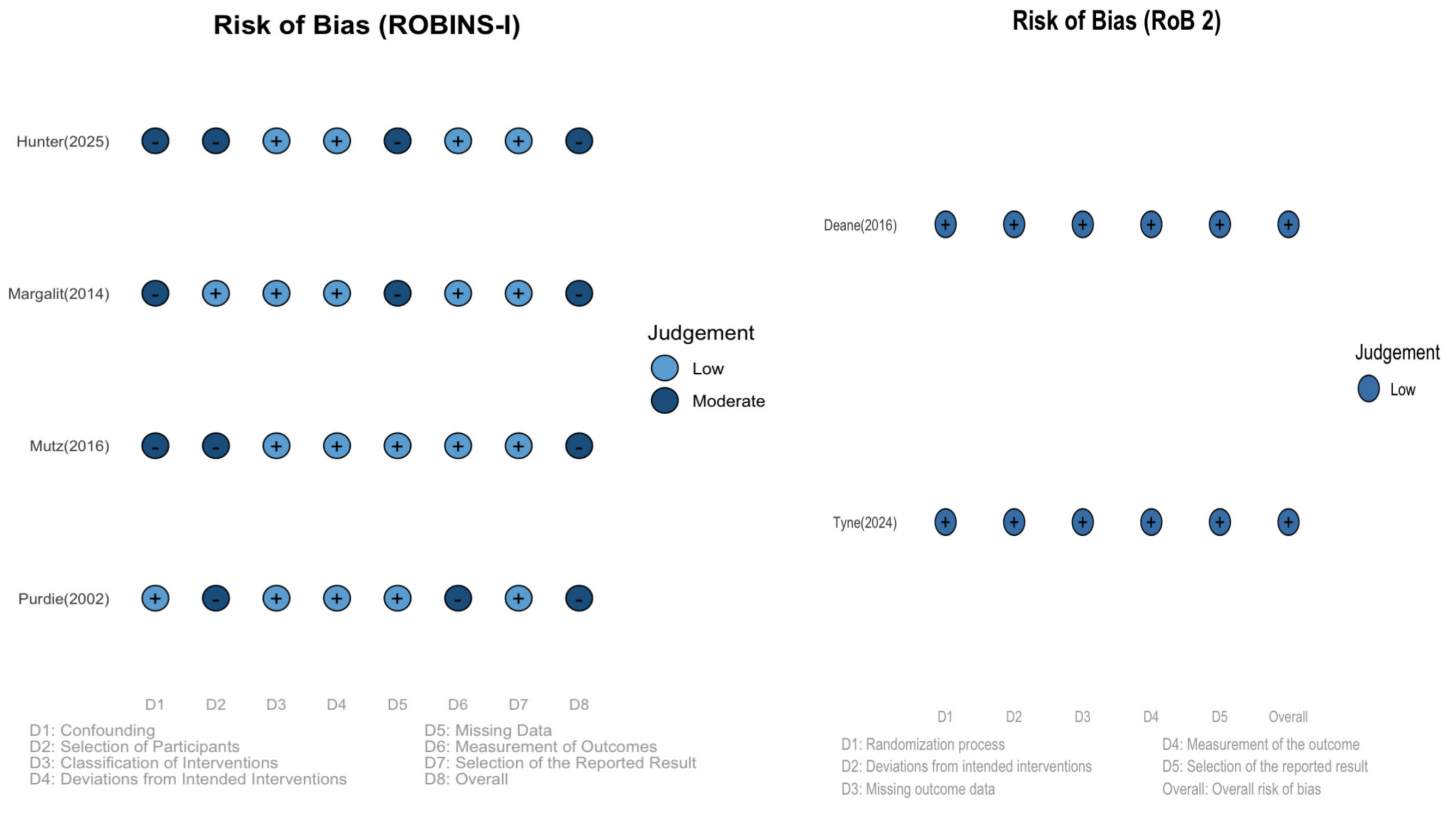
Risk-of-bias assessment of the included studies using design-specific tools (ROBINS-I for non-randomized studies and RoB 2 for randomized controlled trials).

The risk-of-bias assessments for the included studies are presented in **Figure 2**. The two randomized controlled trials assessed using the RoB 2 tool were judged to have an overall low risk of bias across all domains. Among the four non-randomized studies evaluated using ROBINS-I, most studies were judged to have low risk of bias in domains related to classification of interventions, deviations from intended interventions, missing data, measurement of outcomes, and selection of the reported result. Moderate risk of bias was more frequently observed in the domains of confounding and selection of participants. This was primarily due to the lack of randomization and limited control for baseline differences or other potential confounding variables in several non-randomized studies. No non-randomized study was judged to have a serious overall risk of bias.

**Figure 3:**
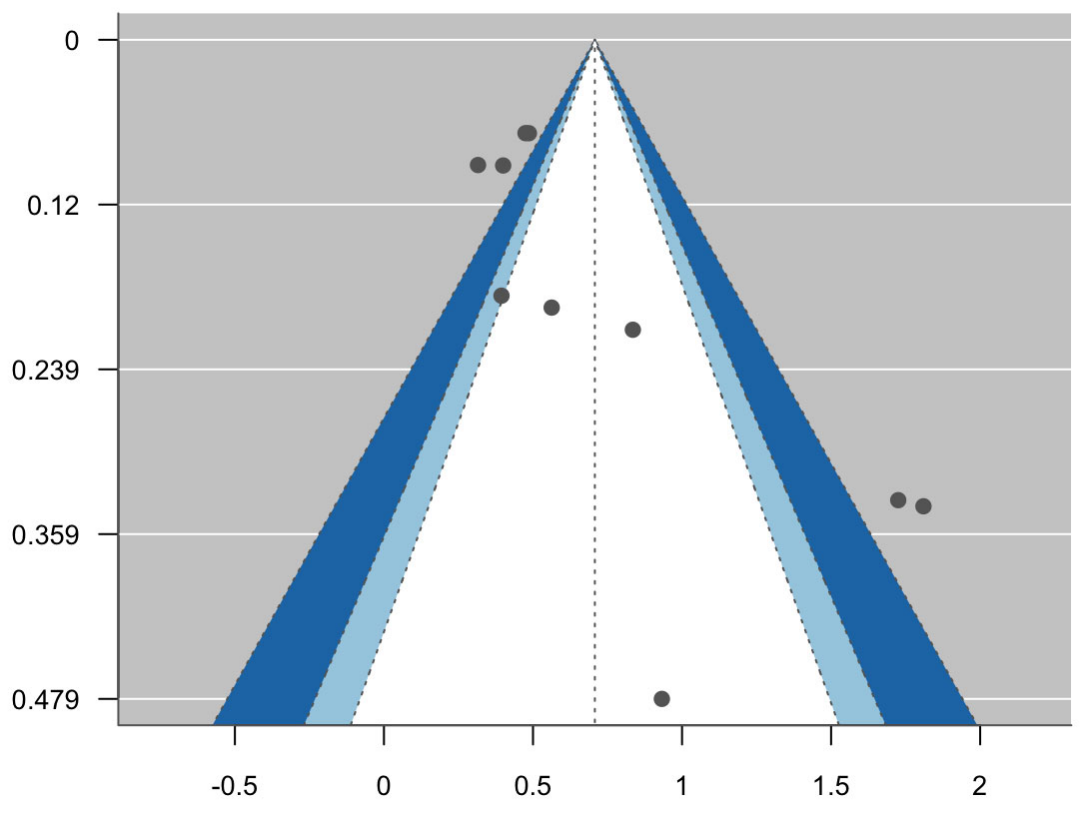
Funnel plot assessing potential publication bias for studies investigating the effect of outdoor exercise interventions on self-efficacy. Visual inspection was used to evaluate asymmetry.

Potential publication bias and small-study effects were examined using Egger’s regression test, and the corresponding funnel plot is presented in **Figure 3**. The test indicated significant funnel plot asymmetry (z = 4.18, p < 0.0001), suggesting the presence of small-study effects. The limit estimate as the standard error approached zero was 0.18 (95% CI −0.03 to 0.38), indicating that the adjusted effect size was attenuated and no longer statistically significant. These findings suggest that the pooled effect may be overestimated and should therefore be interpreted with caution.

Sensitivity analyses were conducted to evaluate the robustness of the findings. Leave-one-out analyses showed that the pooled effect remained statistically significant after sequential exclusion of each study, with effect size estimates ranging from 0.51 to 0.90. In addition, an alternative random-effects model including only one effect size per study yielded a pooled effect size of 0.77 (95% CI: 0.39–1.16, p < 0.001), which was consistent with the primary three-level meta-analysis. These findings suggest that the overall results were not driven by any single study or by the inclusion of multiple effect sizes from individual studies.

### Main effect results

#### Effects of outdoor and nature-based interventions on self-efficacy

**Figure 4:**
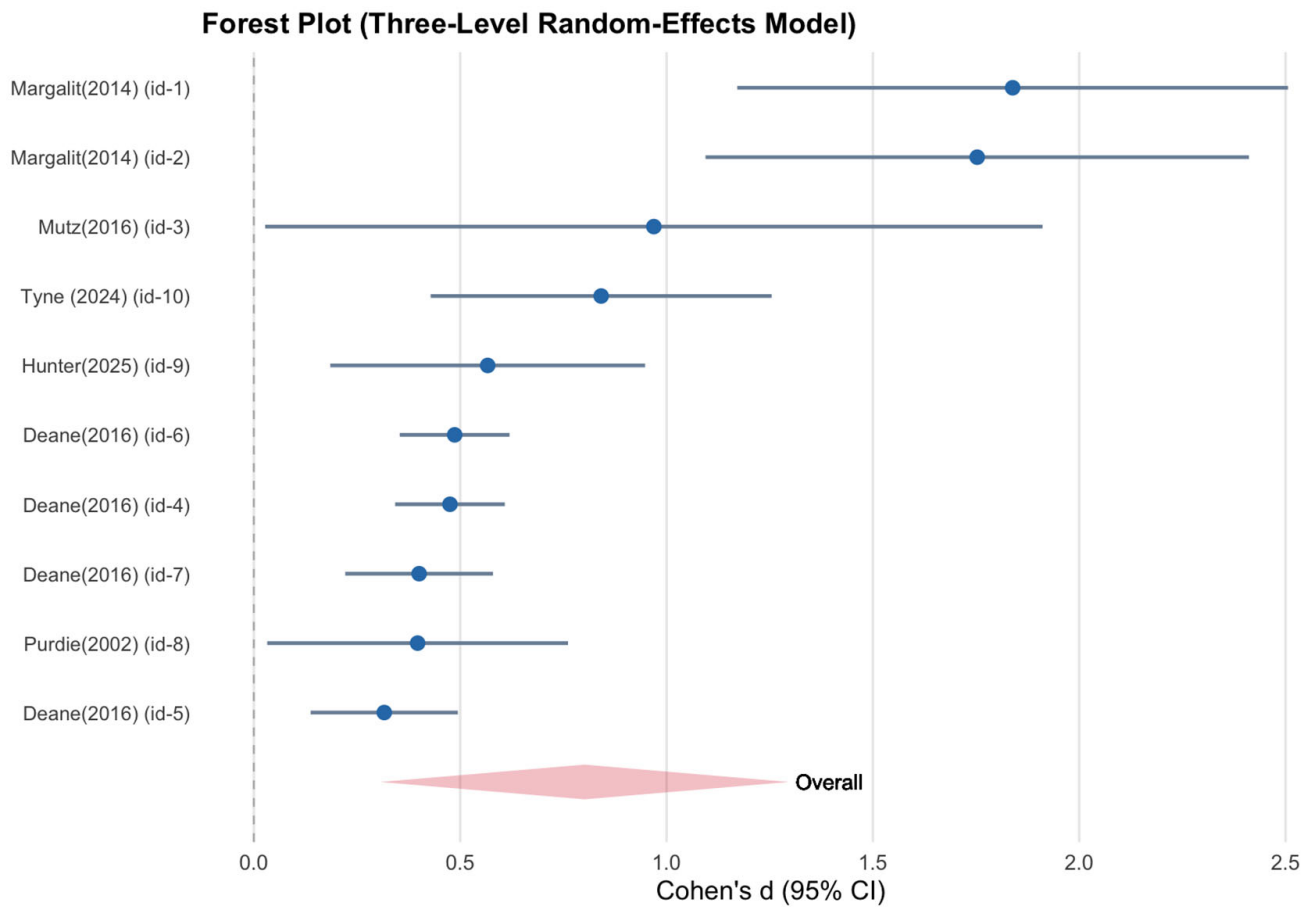
Forest plot of the pooled effects of outdoor exercise interventions on self-efficacy. Effect sizes are expressed as standardized mean differences, with individual study estimates and the overall pooled estimate presented with 95% confidence intervals.

A three-level multivariate meta-analysis including 10 effect sizes from 6 independent studies demonstrated a statistically significant positive effect of Outdoor and Nature-Based Interventions on self-efficacy (d = 0.80, 95% CI 0.31 to 1.30, p = 0.005). Significant heterogeneity was observed across effect sizes (Q = 38.66, p < 0.0001; total I² = 84.8%). Variance decomposition indicated that heterogeneity was primarily attributable to between-study variability (τ² = 0.23), whereas within-study variance was negligible (τ²= 0). These findings suggest that Outdoor and Nature-Based Interventions is associated with improved self-efficacy. However, the results should be interpreted with caution given the relatively small number of studies and the presence of substantial between-study heterogeneity. Detailed results are presented in **Figure 4**.

#### Effects of intervention duration as a moderator

**Figure 5:**
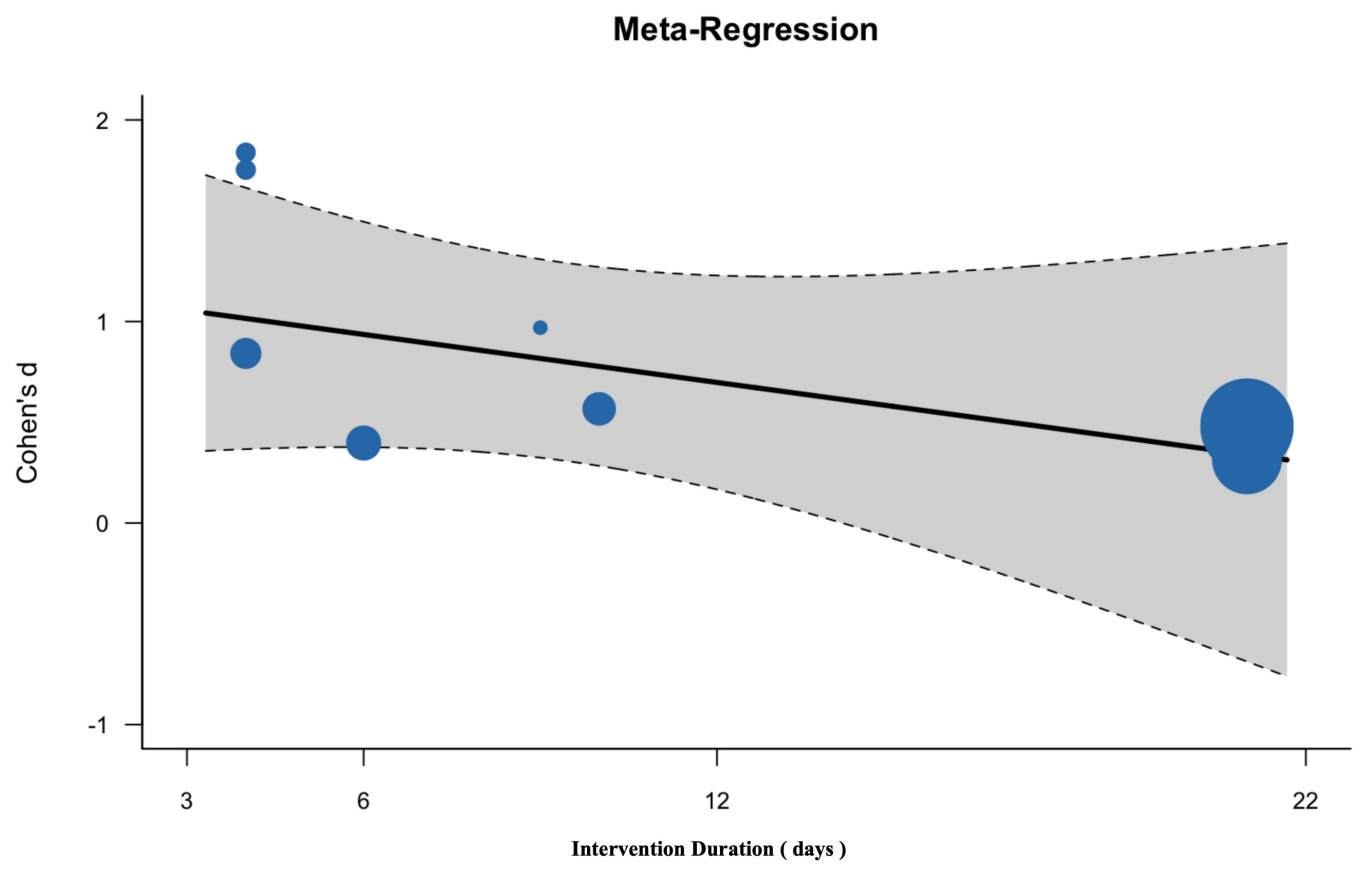
Meta-regression examining the relationship between intervention duration (days) and self-efficacy effect sizes.

The meta-regression analysis examining intervention duration as a moderator showed no statistically significant effect on self-efficacy outcomes (β = −0.040, 95% CI −0.118 to 0.038, p = 0.274). This indicates that the magnitude of the association between outdoor exercise and self-efficacy did not vary significantly with increasing intervention duration. Within the range of durations examined, longer exposure to Outdoor and Nature-Based Interventions was not associated with greater improvements in self-efficacy. Detailed results are presented in **Figure 5**.

### Effects of population as a moderator

**Figure 6:**
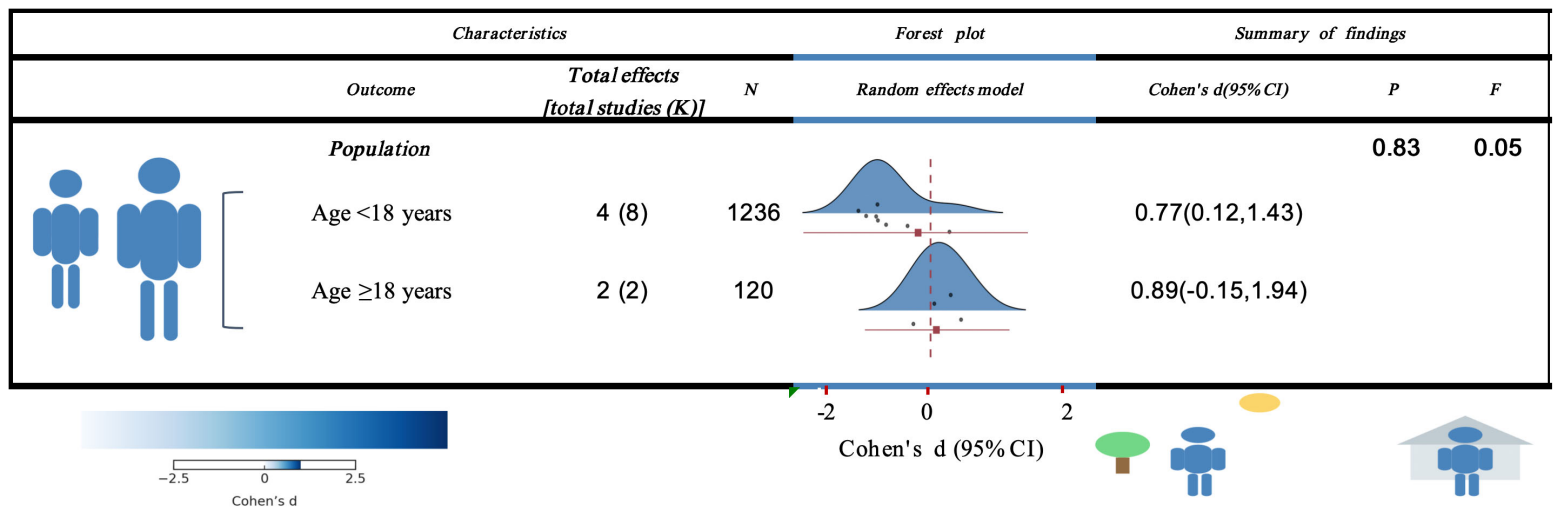
Moderator analysis of the effects of outdoor and nature-based interventions on self-efficacy according to population type (mean age <18 years vs. ≥18 years).

We found no significant differences when considering the moderating effect of population type (F = 0.05, p = 0.83). Specifically, the meta-regression coefficient for participants with a mean age ≥18 years relative to those with a mean age <18 years was not statistically significant (β = 0.12, 95% CI −1.12 to 1.35, p = 0.83). These findings suggest that no significant moderating effect of population type was observed within the available evidence base. However, given the limited number of included studies and effect sizes, this moderator analysis should be considered exploratory and interpreted with caution. Detailed results are presented in **Figure 6**.

## Discussion

This three-level meta-analysis provides a comprehensive synthesis of the existing evidence on the relationship between outdoor and nature-based interventions and self-efficacy and related perceived effectiveness constructs among adolescents and young adults. Importantly, the use of a three-level meta-analytic approach enabled the inclusion of multiple, statistically dependent effect sizes from individual studies, thereby offering a more nuanced and comprehensive synthesis than traditional meta-analyses. Based on 10 effect sizes derived from six independent studies, the findings indicate a large positive association between participation in outdoor and nature-based interventions and self-efficacy and related perceived effectiveness constructs. This result suggests that engagement in outdoor and nature-based interventions is generally associated with meaningful improvements in individuals’ confidence in their ability to manage challenges and perform goal-directed behaviors during critical developmental periods. However, these findings should be interpreted with caution given the limited number of included studies and the substantial heterogeneity observed across effect sizes.

### Effect of outdoor and nature-based interventions on self-efficacy

Our analysis demonstrated a positive effect of outdoor and nature-based interventions on self-efficacy and related perceived effectiveness constructs among adolescents and young adults, indicating that participation in such interventions is generally associated with enhanced confidence in one’s ability to cope with challenges and perform goal-directed behaviors. This finding is consistent with a substantial body of literature suggesting that outdoor and adventure-based programs can promote positive psychosocial outcomes, including self-concept, personal development, perceived competence, and self-efficacy (48, 49). It is also supported by primary studies reporting improvements in self-efficacy following participation in outdoor and nature-based interventions. For example, improvements in outdoor recreation self-efficacy and transfer to academic self-efficacy have been observed following participation in adventure recreation programs (50). Similarly, significant gains in self-efficacy have been reported among at-risk adolescents following participation in wilderness therapy interventions (13).

Several mechanisms may help explain these positive effects. Previous research has suggested that outdoor and adventure-based programs provide opportunities for mastery experiences, progressive challenges, problem-solving, autonomy, and successful task completion, all of which may contribute to enhanced perceptions of competence and personal effectiveness (51).In addition, the novel and immersive nature of outdoor environments may foster emotional engagement, psychological restoration, and adaptive coping (52), thereby reinforcing confidence in one’s ability to manage challenges. Taken together, the consistency between the present findings, previous empirical evidence, and established theoretical explanations suggests that outdoor and nature-based interventions may provide a supportive context for the development of self-efficacy during adolescence and young adulthood.

### Intervention duration as a moderator

Our moderator analysis did not identify a significant association between intervention duration and the magnitude of self-efficacy outcomes. This finding is partially consistent with previous research, which has produced mixed results regarding the role of intervention duration or exposure time. Evidence from related research on green exercise suggests that measurable mental-health benefits can occur even at relatively short exposure durations and did not support a simple “more time = proportionally more benefit” pattern (26). In contrast, a systematic review reported that the most effective nature-based interventions were typically delivered over 8 to 12 weeks, with an optimal session dose ranging from approximately 20 to 90 minutes for improving mental and physical health outcomes (27). Taken together, these findings suggest that the relationship between the duration of outdoor and nature-based interventions and psychological outcomes may not be linear. However, the absence of a significant moderating effect of intervention duration should be interpreted cautiously given the limited number of studies and effect sizes available for analysis. One possible explanation is that qualitative aspects of outdoor and nature-based interventions, such as perceived challenge, successful task completion, and emotional engagement, may contribute to self-efficacy outcomes. Further research is needed to clarify the relative importance of intervention duration and experiential characteristics.

### Population as a moderator

We did not observe a significant moderating effect of population type on self-efficacy outcomes. However, this finding should be interpreted cautiously because the analysis was based on a limited number of studies and effect sizes. This finding is consistent with prior literature suggesting that self-efficacy remains malleable across adolescence and early adulthood and can be influenced by experiential factors in both age groups (53). For instance, previous studies have documented improvements in self-efficacy following outdoor and nature-based interventions among adolescents (13) as well as among university-aged young adults (41). Although the available evidence did not provide evidence of differential effects between age groups, further research is needed to determine whether intervention effects vary across developmental stages.

### Implications for practice and research

The present findings provide several practical implications. First, the overall positive effect observed across diverse intervention types suggests that outdoor and nature-based interventions may represent a promising approach for enhancing self-efficacy among adolescents and young adults. Second, no significant moderating effect of population type was observed; however, this finding should be interpreted cautiously given the limited evidence base. These findings suggest that outdoor and nature-based programs may have potential applications in school curricula, youth development programs, and early adulthood interventions, although further evidence is needed. Finally, the findings suggest that key elements of outdoor and nature-based interventions—such as structured activities, opportunities for successful task completion, and social interaction—may play an important role in enhancing self-efficacy.

### Limitations and future directions

Several limitations of the present meta-analysis should be acknowledged. First, although a three-level meta-analytic approach was employed to appropriately account for statistical dependence among multiple effect sizes, the overall number of included studies was relatively small. This limited sample size may have reduced the statistical power to detect moderator effects and constrained the generalizability of the findings. In addition, publication-bias assessments indicated potential funnel plot asymmetry and small-study effects. However, these methods are known to have limited statistical power and stability when applied to a small number of studies and effect sizes. Therefore, both the pooled effect estimate and the bias-adjusted estimate should be interpreted cautiously. Furthermore, effect sizes were calculated using post-intervention standardized mean differences. Although this approach facilitated consistent effect-size estimation across studies, post-intervention differences in non-randomized studies may still be influenced by baseline imbalance or residual confounding. Therefore, the pooled estimates should be interpreted with appropriate caution. Future research would benefit from a larger body of primary studies examining outdoor and nature-based interventions and self-efficacy, which would allow for more robust subgroup and moderator analyses.

Second, substantial heterogeneity was observed across effect sizes, with most of the variability attributable to between-study differences. While the three-level model enabled variance decomposition, the available data did not permit a detailed examination of several potentially important sources of heterogeneity, such as intervention intensity, program structure, group composition, or specific experiential components (e.g., challenge level, social interaction, or facilitation style). In addition, the included studies used different instruments to assess self-efficacy and related constructs, including general self-efficacy, personal effectiveness, and coping-related efficacy. Although these measures were considered conceptually related and were synthesized using standardized effect sizes, differences in construct coverage may have contributed to heterogeneity across studies. Future studies should report intervention characteristics in greater detail and adopt more standardized reporting practices to facilitate more refined analyses of intervention features.

Third, the operationalization of outdoor and nature-based interventions varied considerably across included studies, wilderness therapy, outdoor education, adventure-based programs, and other structured outdoor activities. Although this diversity enhances ecological validity, it also limits the ability to draw conclusions about the relative effectiveness of specific types of outdoor exercise. Future research should aim to compare different outdoor exercise modalities using well-defined intervention protocols to clarify which program characteristics are most strongly associated with improvements in self-efficacy.

Finally, most included studies relied on short-term post-intervention assessments, limiting conclusions regarding the durability of intervention effects. Given that self-efficacy is a dynamic construct that may evolve over time, future research should incorporate longer follow-up periods and longitudinal designs to examine whether gains in self-efficacy are maintained and whether they translate into sustained behavioral and psychological outcomes.

## Conclusion

Outdoor and nature-based interventions were associated with improvements in self-efficacy and closely related perceived effectiveness constructs among adolescents and young adults. No significant moderating effects were observed for age group or intervention duration, although these findings should be interpreted cautiously given the limited evidence base. These findings suggest that outdoor and nature-based interventions may represent a promising approach for supporting self-efficacy during critical developmental periods. However, the findings should also be interpreted in light of the small number of included studies and indications of potential small-study effects.

## Data Availability

The original contributions presented in the study are included in the article/**Supplementary Material**. Further inquiries can be directed to the corresponding author.
