# Supplementary material for: Impact of outdoor and nature-based interventions on self-efficacy among adolescents and young adults: a three-level meta-analysis
Source: Front Psychiatry. 2026 Jul 14;17:1856344. doi: 10.3389/fpsyt.2026.1856344 (PMC13410772; doi:10.3389/fpsyt.2026.1856344)
Supplement: Supplementary file 1 [file Table1.docx]

**Appendix 1：search strategy**

| **Database** | **Retrieval strategy** |
| --- | --- |
| **Cochranne** | #1 Outdoor and nature-based intervention terms  ("outdoor education" OR "outdoor learning" OR "adventure education" OR (adventure NEXT program*) OR "experiential learning" OR (wilderness NEXT program*) OR (wilderness NEXT therap*) OR "nature-based education" OR (forest NEXT school*) OR "outward bound" OR ((outdoor OR nature OR wilderness) AND (education OR learning OR program* OR camp* OR intervention* OR curriculum OR course* OR expedition* OR adventure))),ab,kw  #2 Population terms  (adolescen* OR teen* OR youth* OR "young people" OR "young adult*" OR "emerging adult*" OR "college student*" OR "university student*" OR undergraduate* OR student* OR "secondary school" OR "high school" OR "middle school"),ab,kw  #3 Self-efficacy terms  ("self-efficacy" OR "self efficacy" OR self-efficac* OR efficacy OR "general self-efficacy" OR "academic self-efficacy"),ab,kw  #4 #1 AND #2 AND #3 |
| **Embase** | #1 Outdoor and nature-based intervention terms  (("outdoor education" OR "outdoor learning" OR "adventure education" OR "adventure program*" OR "experiential learning" OR "wilderness program*" OR "wilderness therap*" OR "nature-based education" OR "forest school*" OR "outward bound"),ab,kw OR ((outdoor OR nature OR wilderness) NEAR/3 (education OR learning OR program* OR camp* OR intervention* OR curriculum OR course* OR expedition* OR adventure)),ab,kw)  #2 Population terms  (adolescen* OR teen* OR youth* OR "young people" OR "young adult*" OR "emerging adult*" OR "college student*" OR "university student*" OR undergraduate* OR student* OR "secondary school" OR "high school" OR "middle school"),ab,kw  #3 Self-efficacy terms  ("self efficacy" OR self-efficac* OR efficacy OR "general self-efficacy" OR "academic self-efficacy"),ab,kw  #4 #1 AND #2 AND #3 |
| **Web of Science** | #1 Outdoor and nature-based intervention terms  TS=(("outdoor education" OR "outdoor learning" OR "adventure education" OR "adventure program*" OR "experiential learning" OR "wilderness program*" OR "wilderness therap*" OR "nature-based education" OR "forest school*" OR "outward bound") OR ((outdoor OR nature OR wilderness) NEAR/3 (education OR learning OR program* OR camp* OR intervention* OR curriculum OR course* OR expedition* OR adventure)))  #2 Population terms  TS=(adolescen* OR teen* OR youth* OR "young people" OR "young adult*" OR "emerging adult*" OR "college student*" OR "university student*" OR undergraduate* OR student* OR "secondary school" OR "high school" OR "middle school")  #3 Self-efficacy terms  TS=("self-efficacy" OR "self efficacy" OR self-efficac* OR efficacy OR "general self-efficacy" OR "academic self-efficacy")  #4 #1 AND #2 AND #3 |
| **PubMed** | #1 Outdoor and nature-based intervention terms  ("outdoor education"[tiab] OR "outdoor learning"[tiab] OR "adventure education"[tiab] OR "adventure program*"[tiab] OR "experiential learning"[tiab] OR "wilderness program*"[tiab] OR "wilderness therap*"[tiab] OR "nature-based education"[tiab] OR "forest school*"[tiab] OR "outward bound"[tiab] OR ((outdoor[tiab] OR nature[tiab] OR wilderness[tiab]) AND (education[tiab] OR learning[tiab] OR program*[tiab] OR camp*[tiab] OR intervention*[tiab] OR curriculum[tiab] OR course*[tiab] OR expedition*[tiab] OR adventure[tiab])))  #2 Population terms  ("Adolescent"[Mesh] OR adolescen*[tiab] OR teen*[tiab] OR youth*[tiab] OR "young people"[tiab] OR "young adult*"[tiab] OR "emerging adult*"[tiab] OR "college student*"[tiab] OR "university student*"[tiab] OR undergraduate*[tiab] OR student*[tiab] OR "secondary school"[tiab] OR "high school"[tiab] OR "middle school"[tiab])  #3 Self-efficacy terms  ("Self Efficacy"[Mesh] OR "self efficacy"[tiab] OR self-efficac*[tiab] OR efficacy[tiab] OR "general self-efficacy"[tiab] OR "academic self-efficacy"[tiab])  #4 #1 AND #2 AND #3 |
| **Sportdiscus** | #1 Outdoor and nature-based intervention terms  ("outdoor education" OR "outdoor learning" OR "adventure education" OR (adventure N1 program*) OR "experiential learning" OR (wilderness N1 program*) OR (wilderness N1 therap*) OR "nature-based education" OR (forest N1 school*) OR "outward bound" OR ((outdoor OR nature OR wilderness) AND (education OR learning OR program* OR camp* OR intervention* OR curriculum OR course* OR expedition* OR adventure)))  #2 Population terms  (adolescen* OR teen* OR youth* OR "young people" OR "young adult*" OR "emerging adult*" OR "college student*" OR "university student*" OR undergraduate* OR student* OR "secondary school" OR "high school" OR "middle school")  #3 Self-efficacy terms  ("self-efficacy" OR "self efficacy" OR self-efficac* OR efficacy OR "general self-efficacy" OR "academic self-efficacy")  #4 #1 AND #2 AND #3 |
| **PsycArticles** | #1 Outdoor and nature-based intervention terms  Any Field: "outdoor education" OR Any Field: "outdoor learning" OR Any Field: "adventure education" OR Any Field: "adventure program*" OR Any Field: "experiential learning" OR Any Field: "wilderness therap*" OR Any Field: "forest school*" OR Any Field: "outdoor program*" OR Any Field: "outward bound" OR Any Field: outdoor OR Any Field: nature OR Any Field: wilderness  #2 Population terms  Any Field: adolescen* OR Any Field: teen* OR Any Field: youth* OR Any Field: "young people" OR Any Field: "young adult*" OR Any Field: "emerging adult*" OR Any Field: "college student*" OR Any Field: "university student*" OR Any Field: undergraduate* OR Any Field: student* OR Any Field: "middle school" OR Any Field: "high school" OR Any Field: "secondary school"  #3 Self-efficacy terms  Any Field: self-efficac* OR Any Field: "self efficacy" OR Any Field: efficacy OR Any Field: "general self-efficacy" OR Any Field: "academic self-efficacy"  #4 #1 AND #2 AND #3 |
